# Phosphorus availability and dynamics in soil affected by long-term ruzigrass cover crop

**DOI:** 10.1016/j.geoderma.2018.09.056

**Published:** 2019-03-01

**Authors:** Danilo S. Almeida, Daniel Menezes-Blackburn, Hao Zhang, Philip M. Haygarth, Ciro A. Rosolem

**Affiliations:** a São Paulo State University, College of Agricultural Sciences, Department of Crop Science, Botucatu 18610-307, Brazil; b Lancaster University, Lancaster Environment Centre, Lancaster LA1 4YQ, UK; c Sultan Qaboos University, Water and Agricultural Engineering, College of Agricultural and Marine Sciences, Department of Soils, PO Box 34, Al-khod 123, Oman

**Keywords:** DET, diffusive equilibrium in thin films, DGT, diffusive gradient in thin films, DIFS, DGT induced fluxes in soils and sediments model, k_−1_, desorption rate constant, K_d_, equilibrium distribution coefficient between solid phase and soil solution, MBP, microbial biomass phosphorus, P_DET_, soil solution P concentration measured by DET, P_DGT_, DGT measured time average P concentration at the interface of soil and DGT device, P_E_, effective P concentration, P_resin_, soil phosphorus extractable with anion exchange pearl resin, R, ratio of P_DGT_ and P_DET_, R_diff_, ratio of P_DGT_ to P_E_ in the case where there is no P resupply from the solid phase, R-R_diff_, relative resupply from solid phase, SOM, soil organic matter, T_c_, response time of (de)sorption process, DGT, DET, *Urochloa ruziziensis*, Fallow, Crop rotation

## Abstract

The use of grasses as cover crops in the off-season of cash crops under no-till has been largely adopted. However, soil phosphorus (P) uptake was previously shown to be reduced when ruzigrass is introduced in the rotation, affecting the viability and sustainability of this cropping system. The objective of this study was to assess the effect of ruzigrass on soil P availability and desorption kinetics under different P fertilizer application rates. A long-term field experiment where soybean (*Glycine* max) has been grown in rotation with ruzigrass (*Urochloa ruziziensis*) or fallow for 10 years, with the application of 0, 13, and 26 kg ha^−1^ of P, was evaluated for two consecutive years. Soil P desorption kinetics was assessed using diffusive equilibrium (DET) and gradient in thin films (DGT) techniques, as well as the DGT-induced fluxes in soils model (DIFS). Microbial biomass P (MBP) was assessed to verify if soil solution P (P_DET_) was reduced due to immobilization by microorganisms. Ruzigrass reduced MBP and P_DET_ especially when P fertilizer was applied. The concentration of labile P (P_DGT_) was also lower after ruzigrass than in fallow. The soil ability to resupply P to soil solution was lower after ruzigrass regardless of P rates due to a slower desorption in response to the perturbation imposed by DGT. Growing ruzigrass as cover crop in the soybean off-season decreases soil P availability regardless of P fertilizer application rates by fundamentally reducing P mobility and P resupply from soil solid phase into soil solution.

## Highlights

• The use of ruzigrass as cover crop affect soil P availability in the long-term.
• Soil P desorption kinetics was assessed using diffusive gradient in thin films (DGT).
• The use of DGT provided new insights into P availability, by considering the P diffusion and resupply process.
• Ruzigrass decreases P availability by reducing P mobility and resupply from soil solid phase into soil solution.

## 1 Introduction

Ruzigrass [*Urochloa ruziziensis* (R. Germ. and C.M. Evrard) Morrone and Zuloaga] has been preferentially used as cover crop in the off-season of cash crops and as forage in integrated crop-livestock systems than other Urochloa grasses, due to its ease of management in desiccation, high yield, palatability, nutritional quality for animal feed, high adaptability to poor phosphorus (P) soils, long residue persistence protecting the soil, and adaptability to climatic conditions during off-season (Boddey et al., 1996; Nascente et al., 2013). These characteristics are very interesting in conservational soil management such as no-till, to increase soil organic matter (SOM), aggregation and biological activity, as well as to protect the soil against erosion, keep soil moisture, and provide an opportunity to increase crop productivity (Castro et al., 2015; Franzluebbers et al., 2014; Lienhard et al., 2012). Additionally, the high P uptake capacity of ruzigrass is important in improving soil P cycling and P use efficiency (Almeida and Rosolem, 2016; Merlin et al., 2015). However, lower yields of cash crops have been observed after growing ruzigrass compared with those in fallowed soil (Almeida et al., 2018c). Almeida et al. (2018c) observed a lower soybean [*Glycine* max (L.) Merrill] grain yield and leaf P concentration after ruzigrass than fallow in four consecutive years. A lower P uptake by maize (*Zea mays* L.) was also observed after ruzigrass than in fallowed soil (Almeida et al., 2018b).

The lower bioavailability of P after growing ruzigrass has been attributed to a possible lower soil P desorption and increased soil P adsorption sites due to interactions of iron (Fe) and aluminum (Al) oxides with SOM (Almeida et al., 2018b). The low soil P bioavailability in soil cultivated with ruzigrass was also showed by a lower depletion of P in the rhizosphere soil of maize, despite the observed increase in P concentration in the bulk soil (Almeida et al., 2018b). Almeida et al. (2018c) found no correlation of plant available P and the estimated P availability by resin method (Raij et al., 1986). Additionally, using Hedley P fractionation (Hedley et al., 1982), Almeida and Rosolem (2016) observed a higher concentration of Resin-, NaHCO_3_-, and NaOH-extractable P, considered labile and moderately labile P fractions, after growing ruzigrass than in fallow. The lack of correlation between standard soil P tests and P uptake by soybean indicates that ruzigrass effect is not well represented by these extraction methods, and other more realistic methods are needed to reflect the plant experience of soil P availability and understand the ruzigrass effect on P mobility and desorption.

Plant P uptake results in P depletion in the rhizosphere, inducing P diffusion towards roots and release of P from soil solid phase (Barber et al., 1963). The diffusive gradient in thin films (DGT) method mimics the plant uptake action, creating a P sink, and inducing P resupply from the soil solid phase to the soil solution (Lehto, 2016). The method of DGT has been successfully used on a wide range of soils to estimate P availability to crops, such as maize (Heidari et al., 2017; Six et al., 2013), barley (*Hordeum vulgare* L.) (Tandy et al., 2011), and wheat (*Triticum aestivum* L.) (Mason et al., 2010). According to Six et al. (2012), the better prediction of soil P availability than conventional P extractions is explained by the diffusional process accounted for by DGT method, the lower anionic interferences with DGT, the non-labile P extracted by shaking and small solid:solution ratios in conventional extractions, and because P determination is unreliable if the extract P concentrations are near the detection limit. The use of an adequate soil analysis to estimate soil P availability not only renders a more precise determination of P bioavailability, but also an accurate diagnosis of the mechanisms behind the data variability, contributing in the search for more sustainable food production systems.

The objective of this study was to test the long term effect of ruzigrass on soil P availability and desorption kinetics at different P fertilizer application rates at field conditions. Specifically, we aimed to test the hypothesis that growing ruzigrass (cover-crop) in the off-season reduces P availability to the soybean (cash-crop) due to reduced soil P desorption rates.

## 2 Material and methods

### 2.1 Experimental site and treatments

A long-term field experiment located in Botucatu, State of São Paulo, Brazil (22°50′00″ S; 48°25′31″ W; and altitude of 806 m), was carried out for two years (2014 and 2015). The soil is a Rhodic Hapludox (Soil Survey Staff, 2014) with 670 g kg^−1^ of sand and 210 g kg^−1^ of clay. The area selected for the experiment has been under no-till since 1998. From 1998 to 2005, all plots received a total of 96 kg ha^−1^ of P as triple superphosphate (TSP), and soybean was cropped in the summer in rotation with black oat (*Avena strigosa*, Schreb.) and triticale (*x Triticosecale*, Witt.) in the fall-winter, and with pearl millet [*Pennisetum glaucum*, (L.) R. Br.] in the spring. From 2006 when the present experiment started, soybean has been grown in rotation with ruzigrass or fallowed, with the application of 0, 13, and 26 kg ha^−1^ of P as triple superphosphate in the soybean seed furrow each year. The experimental design was a 2 × 3 factorial in randomized complete block design, with four replications. Plots were 8.0 m long × 5.0 m wide. In the fallow treatment, weed growth was random and sparse, with insignificant dry matter production by the end of the off-season period.

Six soil subsamples were randomly collected per plot to compose one sample per depth per field replication, using a 50 mm diameter core sampler, at depths of 0–5, 5–10, 10–20 and 20–40 cm, in November 2014 and 2015 after ruzigrass desiccation. The samples were air-dried and passed through a 2-mm sieve for subsequent chemical analysis.

### 2.2 Soil chemical characterization

Selected chemical characteristics of the soil of the experimental area are presented in Table 1. Soil pH was determined in a 0.01 mol L^−1^ calcium chloride (CaCl_2_) suspension (1:2.5 soil/solution), potential acidity (H + Al) at pH 7.0 was estimated by the Shoemaker-McLean-Pratt (SMP) pH buffer method (Shoemaker et al., 1961), and cation exchange capacity (CEC) was calculated as the sum of (H + Al) + Ca + Mg + K. The soil labile P (P_resin_) and exchangeable Ca, Mg, and K were extracted with a mixture of anionic resin (IRA-400) and cationic resin (IR-120), following method described by Raij et al. (1986). The resin method was used here since it is the official method to estimate labile P in São Paulo state. The resins were converted to the HCO_3_-form using a solution of 0.1 mol L^−1^ sodium bicarbonate (NAHCO_3_), pH 8.5. Briefly, 2.5 cm^3^ of air-dried soil and a glass marble about 1.8 cm of diameter were added to 25 mL of deionized water. The glass marble was used to complete disaggregate the soil, shaking on an orbital shaker for 15 min at 220 rpm. The glass marble was then removed, and 2.5 cm^3^ of the resin mixture was added, and the samples were shaken overnight for 16 h at 220 rpm. The separation of the resin from the soil was performed by sieving, using a 0.4-mm sieve. The resin was eluted and shaken in 50 mL of a solution of 0.2 mol L^−1^ hydrochloric acid (HCl) and 0.8 mol L^−1^ ammonium chloride (NH_4_Cl) to extract P, Ca, Mg, and K from the resin. The P concentration was determined according to Murphy and Riley (1962) at absorbance of 885 nm using a spectrophotometer UVmini-1240 (Shimadzu Scientific Instruments, Japan), and the Ca, Mg, and K concentrations were determined using an atomic absorption spectrometer AAS7000 (Shimadzu Scientific Instruments, Japan).

**Table 1:** Selected chemical characteristics of soil, at four soil depths, for each treatment, with ruzigrass or fallow, and with 0, 13, or 26 kg ha^−1^ of P, in 2014 and 2015.

Depth	pH^a^	P_resin_^b^	H + Al^c^	CEC^d^	Depth	pH^a^	P_resin_^b^	H + Al^c^	CEC^d^
cm		mg dm^−3^	mmol_c_ dm^−3^		cm		mg dm^−3^	mmol_c_ dm^−3^	
Ruzigrass					Fallow				
2014									
0 kg ha^−1^ of P									
0–5	6.3	13	13	56	0–5	6.2	15	13	56
5–10	5.1	5	19	41	5–10	5.7	5	17	43
10–20	4.8	6	27	36	10–20	4.8	5	28	39
20–40	4.4	3	35	42	20–40	4.2	3	44	50
13 kg ha^−1^ of P									
0–5	6.4	19	12	61	0–5	6.3	18	13	53
5–10	5.7	7	18	45	5–10	5.6	9	18	43
10–20	4.6	5	30	41	10–20	4.7	6	27	39
20–40	4.2	3	39	44	20–40	4.2	3	39	45
26 kg ha^−1^ of P									
0–5	6.3	26	13	52	0–5	6.4	26	12	60
5–10	5.5	18	22	47	5–10	5.9	13	16	44
10–20	4.5	7	34	45	10–20	4.8	7	27	39
20–40	4.2	4	39	44	20–40	4.3	3	39	44

2015									
0 kg ha^−1^ of P									
0–5	6.5	13	13	60	0–5	6.7	15	13	57
5–10	6.0	8	18	48	5–10	7.5	7	15	44
10–20	5.4	7	24	39	10–20	5.8	8	25	40
20–40	5.0	3	34	44	20–40	5.2	3	40	49
13 kg ha^−1^ of P									
0–5	7.1	22	12	64	0–5	6.7	20	13	61
5–10	6.6	8	18	49	5–10	6.2	10	19	47
10–20	6.1	6	28	43	10–20	5.2	4	28	42
20–40	5.6	3	41	48	20–40	4.9	3	35	44
26 kg ha^−1^ of P									
0–5	6.6	32	14	68	0–5	6.7	24	14	57
5–10	6.1	27	22	51	5–10	6.5	20	18	48
10–20	5.5	14	31	47	10–20	6.1	8	26	42
20–40	5.2	2	38	45	20–40	5.7	3	35	42

a Soil pH measured in calcium chloride solution.

b Phosphorus extracted with pearl resin.

c Potential acidity.

d Cation exchange capacity.

### 2.3 Diffusive gradient and equilibrium in thin films

**Fig. 1:**
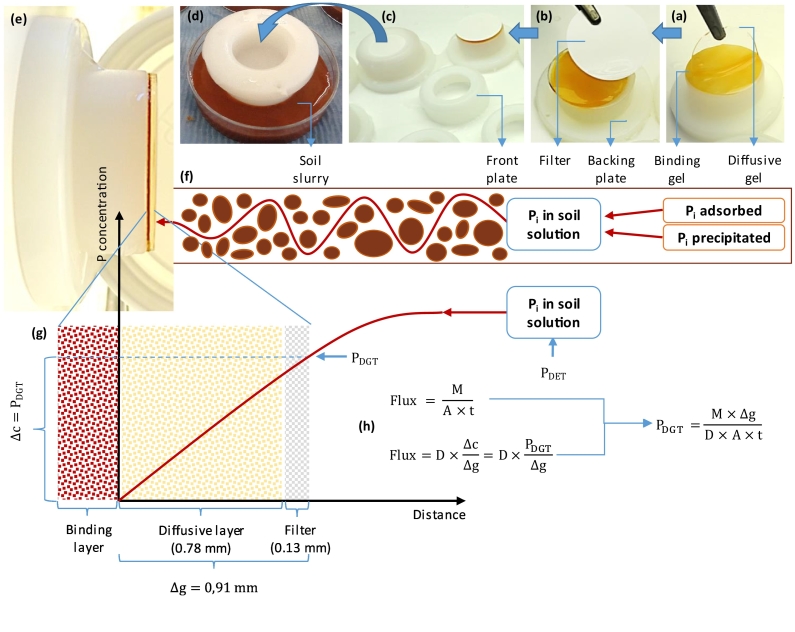
Schematic DGT device assembling (a, b and c) and deployment in soils (d), lateral view of the DGT device (e), a schematic of soil phosphorus (P) desorption/diffusion towards the DGT surface (f), a graphic representation of the P concentration gradient inside and outside the DGT device (g) based on Zhang and Davison (1995). The inorganic P (P_i_) concentration is represented as a function of the distance to binding layer. The concentration of P_i_ at the interface between binding and diffusive layer is effectively zero, due to the rapidly and irreversible binding of P, accounting for the accumulated P mass (M) in the binding layer. The equations used to calculate P_DGT_ using the experimentally measured P flux and Fick's law of diffusion are also illustrated (h), where: A is the exposure area of the DGT, t is the deployment time, D is the diffusive coefficient of the diffusive layer and Δg is the thickness of the diffusive layer.

Soil labile P was measured using diffusive gradient in thin films (DGT) and soil solution P was measured using diffusive equilibrium in thin films (DET) as in Menezes-Blackburn et al. (2016b). A DGT device consists of two plastic plates, a backing plate and a front plate with an exposure window (area = 2.54 cm^2^) (Fig. 1). Between the two plastic plates it contains a binding gel layer, a diffusive gel layer, and a filter membrane. The binding layer (0.60 mm thickness) were placed at the back of the diffusive layer (0.78 mm thickness), and a 0.13 mm thick polyether sulfone filter (0.45 μm) was placed on top of the diffusive layer. The DET devices contain only the diffusive layer and the filter, packed into plastic plates similar to the ones used for DGTs. Gel solution containing acrylamide cross-linkers (DGT Research Ltd., Lancaster, UK) was used to prepare the diffusive and binding layers. To prepare the gels, ammonium persulfate (70 μL, 10% w/v) and 20 μL of TEMED catalyst were added to 10 mL of gel solution, and cast between two glass plates separated by a plastic spacer and allowed to polymerize. In order to prepare the binding layer, gels were soaked in 0.1 mmol L^−1^ FeCl_3_ solution, as in Santner et al. (2010). After cutting the gel, each piece of gel disc was placed in a freshly prepared buffer pH 6.7, MES (4-morpholinoethanesulfonic acid hydrate), 0.05 mol L^−1^, for 2 h. The binding gels were then washed 3 times (2-h intervals) with ultra-pure Milli-Q (MQ) water (Millipore).

Samples of 80 g of dried soil were placed in a container and wetted with MQ water to approximately 50% of maximum water holding capacity to re-establish microbial activity, and incubated for 72 h at 24–25 °C. After incubation, the soil slurry was prepared by adding MQ water and mixing the soil until maximum retention was reached (Zhang et al., 1998). The DET devices were deployed in the soil slurry for 24 h. On the next day, the DGT devices were deployed at the same conditions, for 48 h. The diffusive gel in DET and binding gel in DGT were eluted in 0.5 mL of H_2_SO_4_ solution, 0.25 mol L^−1^. The DGT and DET devices were deployed in duplicates for each experimental replicate. The P concentration in the eluent was measured using the malachite green method (Van Veldhoven and Mannaerts, 1987). The malachite green method measures the inorganic P, with the advantages of its simplicity, stability of reagents, and higher sensitivity than the commonly used Murphy-Riley method. Basically, the malachite green method is based on the malachite green-phosphomolybdate complex formed under acidic conditions, by the reaction of the inorganic P with ammonium molybdate [(NH_4_)_6_Mo_7_O_24_)-4H_2_O] and then with the color obtained after addition of malachite green oxalate (C_52_H_54_N_4_O_12_). The molybdate reactive P (P_i_) was read on a Multiskan spectrophotometer (Thermo Fisher Scientific Inc.,UK).

The concentration of P in the diffusive layer of DET devices (P_DET_) is expressed as the equilibrium concentration to soil solution P. The concentration of labile P (P_DGT_) at the surface of the DGT devices was calculated using Eq. (1) (Zhang and Davison, 1995).

(1) $$P_{\mathrm{DGT}}=\frac{\mathrm{M∆g}}{\mathrm{DAt}}$$

where M is the accumulated P mass in the binding layer, A is the surface area of the DGT sampling window (2.54 cm^2^), t is the deployment time, Δg is the total thickness of the diffusive gel layer and the filter membrane, and D is the diffusion coefficient of P in the diffusive gel. Fig. 1 contains a brief explanation about how the DGT equation was obtained. More information about the principles behind DGT are published in Davison and Zhang (2016).

The ratio of measured labile P concentration, P_DGT_, to soil solution concentration measured by DET, P_DET_, expressed as R was calculated as in Eq. (2).

(2) $$R=\frac{P_{\mathrm{DGT}}}{P_{\mathrm{DET}}}$$

The diffusive only ratio (R_diff_) between P_DGT_ and soil solution P (P_DET_) was calculated using a dynamic numerical model of the DGT-soil system (DIFS model, Harper et al., 2000). It represents the case where there is no resupply from the solid phase and the only supply of P to the DGT (a sink) is from the diffusion in soil solution. Input parameters of particle concentration (ratio between dry and soil solution), soil porosity, and the diffusion coefficient of P in soils were calculated as described by Harper et al. (1998). To calculate R_diff_, the “diffusion only” condition was simulated setting the system response time (T_c_) artificially to 1 × 10^10^ s (extremely slow response) and K_d_ as 10^−10^ cm^3^ g^−1^ (extremely small labile solid phase pool). The relative resupply from solid phase (R-R_diff_) was calculated subtracting the R_diff_ from the R ratio.

The P_DGT_ could be converted to an effective concentration (P_E_) using eq. 3 (Zhang et al., 2001), to represent the P available from both the soil solution and the solid-phase labile pool.

(3) $$P_{E}=\frac{P_{\mathrm{DGT}}}{R_{\text{diff}}}$$

Assuming that P_resin_ provides an estimate of the labile solid phase pool, the partition coefficient between P in the solid and solution phases (K_d_) was calculated as P_resin_/P_DGT_. Using the DIFS model, the T_c_ was obtained, thereby the desorption rate constants (k_−1_) can be estimated as in Eq. (4) (Harper et al., 1998).

(4) $$k_{-1}=\frac{1}{T_{c}\;\left(1+K_{d}P_{c}\right)}$$

### 2.4 Microbial biomass phosphorus

The DGT analysis was performed in non-sterile soil in order to better represent the field conditions. Therefore, some of the desorbed P from the soil solid phase can be intercepted and locked (immobilized) in the microbial biomass before it accumulates on the DGT. The microbial biomass P (MBP) was simultaneously assessed in order to understand if the observed changes on P mobility and availability were affected by P immobilization by the soil microbial community. The MBP was measured according to McLaughlin et al. (1986) with modifications proposed by Stutter et al. (2015), using the same soil slurry prepared for DGT and DET analysis. Quadruplicates of soil slurry (1 g of dry weight equivalent) were extracted for 16 h in 10 mL of MQ water with anion exchange resin strips either with or without addition of 0.4 mL hexanol. After 16 h, the resins were eluted with 0.5 mol L^−1^ HCl and the concentration of P was measured by the malachite green method. The MBP was estimated as the difference between samples extracted with and without hexanol. A correction factor to account for sorption of P to soil solid phase during extraction was determined from soil samples spiked with 20 mg g^−1^ of P immediately before adding the resin strips.

### 2.5 Statistical analysis

Data from each soil layer were subjected to analysis of variance using a general linear model (Proc GLM) in SAS software (version 9.4, SAS Inst., North Carolina, US), considering a 2 × 3 factorial in randomized complete block design, with four replications. When the F test was significant (*p* < 0.05), treatment means were compared by Student's *t*-test (*p* < 0.05).

## 3 Results and discussion

**Fig. 2:**
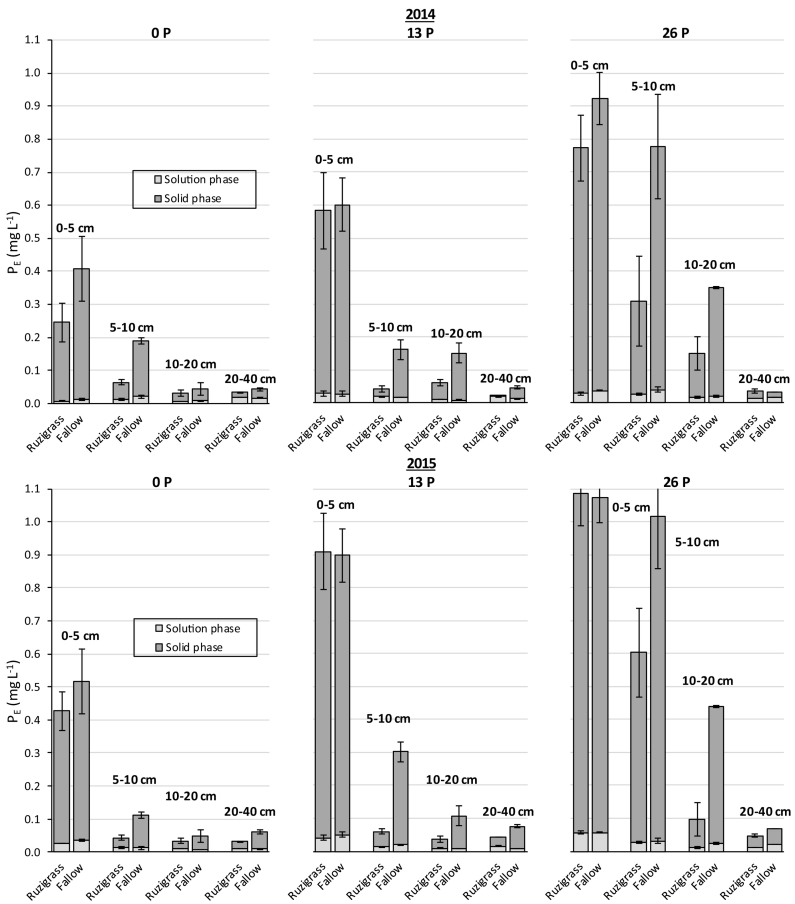
Effective phosphorus concentration (P_E_) in soils as affected by ruzigrass or fallow and P rates (0, 13, and 26 kg ha^−1^) in four soil depths in 2014 and 2015. The soil solution phase (P_DET_) and solid phase (P_E_ − P_DET_) contributions are detailed in light and dark gray, respectively. Error bars represent the standard error.

The labile P accumulation next to soil surface in agricultural systems under no-till was reflected in the higher P_DET_ (Table S1) and P_DGT_ (Table 2) concentrations, for all treatments. According to Pavinato et al. (2009), the stratification of P distribution in the soil profile under no-till is accentuated due to the minimal soil disturbance compared with conventional systems, as well as to the application of phosphate fertilizers and decomposition of plant residues at the soil surface, and to the very low P mobility in soils. In average, the P_DET_ values were 75% lower in the 20–40 cm of soil depth than in the 0–5 cm, while the P_DGT_ in the soil profile was 95% lower in the 20–40 cm depth than the uppermost soil layer, indicating an expected stronger P fixation and lower P bioavailability in 20–40 cm than in the topsoil. The lower P_DGT_ in the deep soil layers compared with the uppermost layers suggests that not only the P supply from the solution phase is lower, but also the P resupply from the solid phase is reduced at depth, as observed in Fig. 2.

It is interesting to notice that the P_DET_ and P_DGT_ concentrations were much lower in the present study than those found by Menezes-Blackburn et al. (2016b) in several UK soils sampled from arable, grassland, and moorland topsoils. The lowest P_DET_ and P_DGT_ concentrations reported by Menezes-Blackburn et al. (2016b) were almost 10 and 4 fold higher than those observed in the current study, respectively. However, the P_DGT_ concentrations determined in the present experiment are within the range found by Six et al. (2014), in weathered soils from Kenya amended with TSP or organic materials, and also similar to the observed by Mason et al. (2010) in some soils from Australia. The differences of the observed P_DGT_ from UK soils is probably due to the high adsorption of P to secondary minerals and precipitation with Fe and Al in our highly weathered and acidic soils (Gérard, 2016; Lindsay, 1979), and also due to the long-term P application resulting in a large P ‘bank’ in UK soils (Menezes-Blackburn et al., 2017), contributing for higher P desorption rates (Shariatmadari et al., 2006).

**Table 2:** Phosphorus concentration measured by DGT devices (P_DGT_) and resupply labile pool size (K_d_) in four soil depths with ruzigrass and different P application rates, in 2014 and 2015.

Off-season	P rates (kg ha^−1^)			Off-season	P rates (kg ha^−1^)		
	0	13	26		0	13	26
2014				2015			
P_DGT_ (μg L^−1^							
0–5 cm							
Ruzigrass	5.1 Bb	12.6 Aa	16.7 Aa	Ruzigrass	9.0 Ab	20.8 Aa	23.4 Aa
Fallow	8.8 Ab	12.6 Ab	19.3 Aa	Fallow	10.9 Ab	17.4 Aa	22.3 Aa
5–10 cm							
Ruzigrass	0.7 Ab	0.8 Ab	5.8 Ba	Ruzigrass	0.9 Ab	1.3 Ab	12.7 Ba
Fallow	1.1 Ab	3.0 Ab	14.5 Aa	Fallow	2.3 Ab	6.3 Ab	20.9 Aa
10–20 cm							
Ruzigrass	1.2 Bb	1.1 Bb	5.2 Aa	Ruzigrass	0.6 Ab	0.6 Bb	1.7 Ba
Fallow	3.3 Ab	2.8 Ab	5.5 Aa	Fallow	0.9 Ac	1.9 Ab	7.9 Aa
20–40 cm							
Ruzigrass	0.8 Aa	0.5 Bb	0.8 Aa	Ruzigrass	0.7 Bb	0.9 Ba	0.9 Ba
Fallow	0.9 Aa	1.0 Aa	0.7 Ab	Fallow	1.4 Ab	1.6 Aa	1.6 Aa

K_d_ (cm^3^ g^−1^)							
0–5 cm							
Ruzigrass	1842 Aa	770 Ab	976 Ab	Ruzigrass	501 ^ns^	583 ^ns^	527 ^ns^
Fallow	1216 Ba	758 Ab	713 Ab	Fallow	508 ^ns^	431 ^ns^	443 ^ns^
5–10 cm							
Ruzigrass	508 Aab	395 Ab	709 Aa	Ruzigrass	633 Ab	711 Ab	1123 Aa
Fallow	293 Ab	530 Aa	349 Bab	Fallow	582 Aa	546 Aa	667 Ba
10–20 cm							
Ruzigrass	1002 Aa	415 Ab	457 Ab	Ruzigrass	1013 Aa	745 Aa	1267 Aa
Fallow	579 Ba	656 Aa	392 Aa	Fallow	1306 Aa	508 Ab	347 Bb
20–40 cm							
Ruzigrass	155 ^ns^	131 ^ns^	250 ^ns^	Ruzigrass	371 Aa	193 Ab	218 Ab
Fallow	200 ^ns^	219 ^ns^	181 ^ns^	Fallow	402 Aa	367 Aa	165 Ab

^a^ Averages followed by different lowercase letters in the line and uppercase in the column are significantly different according to *t*-test (*p* < 0.05). ^ns^ Not significant.

The differences between soil cultivated with ruzigrass and fallow were observed mainly in depths below 5 cm. The ruzigrass has resulted in P immobilization and decreases the soil solution concentration, as observed in the P_DET_ analysis (Table S1). The P_DET_ concentration was lower after ruzigrass than fallow at 5–10 cm of soil depth when 26 kg ha^−1^ of P was applied. In 2015, the P_DET_ concentration at 10–20 and 20–40 cm depth was lower only after ruzigrass than fallow when 26 kg ha^−1^ of P was applied. The P_DGT_ concentration was also lower after ruzigrass than fallow below 5 cm depth, in both years (Table 2). According to (Almeida et al., 2018a), ruzigrass is responsive to P application, with a higher P uptake when it is fertilized, probably due to a more extensive root system, able to reach great soil depths. This could consequently result in decreased P_DET_ even at deeper soil layers, as observed at 20–40 cm depth in 2015. Depletion of P in deep soil layers have been observed after ruzigrass, which could be a strategy to cycle P from deeper soil into uppermost soil layers (Almeida and Rosolem, 2016). However, no increase of P_DET_ or P_DGT_ was observed in the uppermost layers after ruzigrass in the present study. According to Fick's first law of diffusion, the flux of P is proportional to the gradient of P concentrations, being highest when P concentration in soil solution is high and next to roots surface is low. Thus, once P_DET_ is reduced, P movement to DGT will be reduced, accounting for a lower P_DGT_ (Lehto, 2016) and a lower P availability. It is worth mentioning that diffusion is the main mechanism by which P moves towards the root surface where it can be taken up (Barber et al., 1963).

The P_DET_ is expressed as the equilibrium concentration of P between soil solution and the DET gel and it was assumed as the soil solution P_i_ concentration. It has been shown that there is no P binding to the gel, even at high P concentrations (1000 μg L^−1^) (Jarvie et al., 2008). Due to the low ratio between the gel volume and the soil solution volume during DET deployment, no significant dilution is expected of soil solution P after equilibrium is reached. (Davison and Zhang, 2012). The use of P_DET_ was briefly assessed experimentally and compared to other methods (centrifugation and vacuum extraction; data not shown) and seemed to be more appropriated since it gave R values at reasonable ranges; while other methods seemed to contain suspended particles and overestimated solution P concentration, often gave unreasonably low R values. The use of DET to minimize solution P estimation artifacts was also discussed by Menezes-Blackburn et al. (2016b). It is also worth mentioning that the species of P measured by DGT and DET should be the same (the same diffusive gel with the same pore sizes and the same analytical method), although the concentrations are different due to depletion of P at the interface of DGT and soil.

The P_E_ is an effective concentration which convert all P supply to DGT device as diffusion only (Zhang et al., 2001). It represents the labile P (solution P plus the desorbed P). The observed P_E_ values were also lower after ruzigrass than in fallow soil below 5 cm depth mainly at 5–10 and 10–20 cm depth, when 26 kg ha^−1^ of P was applied, in 2014, and at 5–10, 10–20, and 20–40 cm depth, when P was applied, in 2015 (Table S1). The proportion of P_E_ resupplied from the soil solid phase (P_E_ − P_DET_) was markedly higher than from the solution phase (P_DET_), and ruzigrass resulted in a lower P resupply from a solid phase below 5 cm depth, in both years, mainly when P was applied (Fig. 2).

**Fig. 3:**
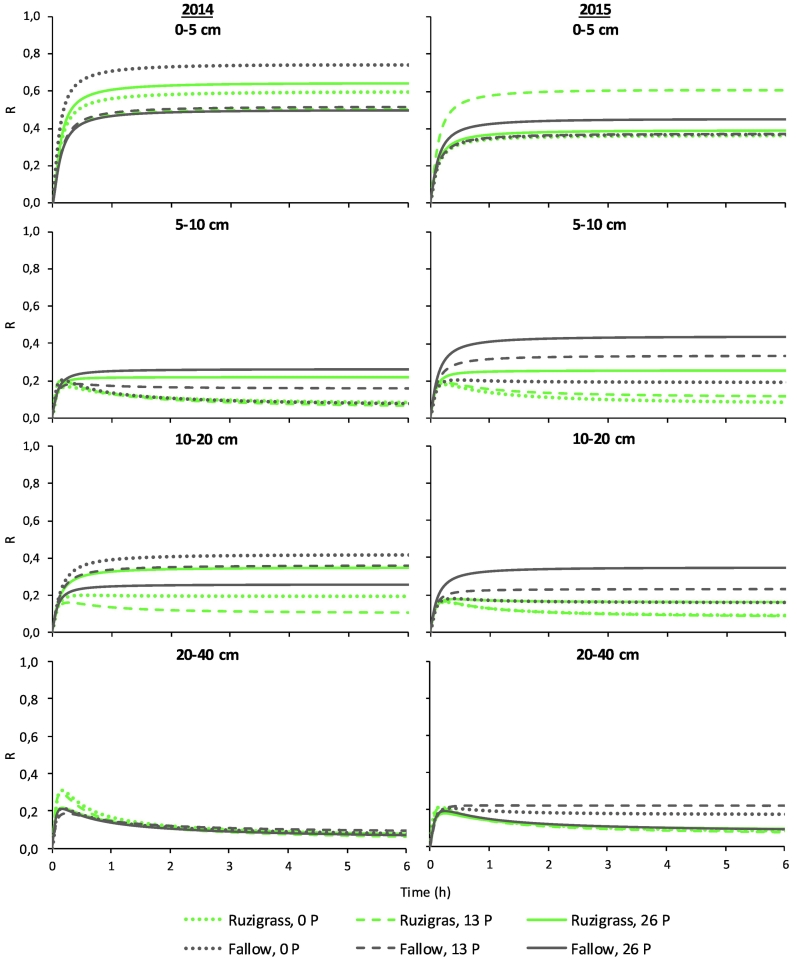
Time dependence of R curves in four soil depths as affected by ruzigrass and P rates (0, 13, and 26 kg ha^−1^), in 2014 and 2015.

According to Menezes-Blackburn et al. (2016b), the labile pool size, K_d_ represents the labile P in the soil solid phase (adsorbed and precipitated P) and soil solution P, accounting for the easily desorbed and readily soluble forms of inorganic P. Interestingly, the K_d_ was higher after ruzigrass than fallow at 0–5 and 10–20 cm in 2014 when P was not applied, and at 5–10 cm in both years, when 26 kg ha^−1^ of P was applied (Table 2). A high K_d_ means a high P resupply potential from the soil solid phase, notwithstanding the real P resupply may be lower than K_d_. These high sustained K_d_ means that either P is being super estimated in the labile soil solid phase resulting in great P_resin_ or the absence of a P mobilization mechanism that could be present on the fallow treatment resulting in lower P concentration in soil solution on the ruzigrass treatment. Almås et al. (2017) showed that high K_d_ indicates that the soil is P deficient, nevertheless, it may as well reflect the overestimation of labile soil solid phase P when using stronger reacting agents. Additionally, Almås et al. (2017) observed that this difference is more visible as total soil P is high, which does not corresponds to conditions in our study where total P is around 380 mg kg^−1^ (Almeida and Rosolem, 2016). The R value is an indicative parameter of the soil ability to supply P to the DGT, a sink of P (Harper et al., 2000). The ratio R of measured P_DGT_ to the P_DET_ was lower after ruzigrass than fallow at the layer of 5–10 cm in 2014, when P was applied, and at 5–10 and 10–20 cm in 2015, regardless of P rates (Table 3). When P was applied at 13 kg ha^−1^ or not applied, the R value was lower after ruzigrass than fallow at 10–20 cm in 2014, and at 20–40 cm in 2014 and 2015. By analyzing the time dependence of R values (Fig. 3), is evident that the P supply to DGT was affected by the use of ruzigrass cover crop, P fertilizer application rates, and soil depths (Fig. 3). According to Zhang et al. (1998), the maximum R value is achieved within the first hour of the DGT deployment (diffusive dominated peak; Fig. 3), and may reach a constant value or decreases progressively. The extension of this decrease is determined by the ability of P resupply from the solid phase (Zhang et al., 1998) and was markedly different among the treatments and soil depths (Fig. 3). In general, the 0–5 cm soil depth had a higher P concentration, which provided conditions for a higher P resupply and to reach a constant P accumulation in the binding layer. This case is assumed as a steady state partial case, characterized by high K_d_ and intermediate T_c_, according to Harper et al. (2000). In the 0–5 cm depth, P supply to DGT was constant after 1 h of deployment for all treatments, pattern that is coherent with a high P resupply from solid phase. On the other hand, in the 5–10 cm depth, there was a pick of P supply to DGT followed by a decrease in the first hour of deployment (Fig. 3), when P was not applied, and at 13 kg ha^−1^ of P when ruzigrass was grown in 2014, while in 2015 the decreased P supply was observed only when ruzigrass was grown with 0 and 13 kg ha^−1^ of P. In the 10–20 cm depth, the decrease of P supply was also observed only when ruzigrass was grown with 0 and 13 kg ha^−1^ of P. When the soil solid phase is not able to resupply P in significant amounts to soil solution, the P in soil solution is depleted and the R ratio would be decreased, reaching the partial non-steady state case, according to Harper et al. (2000). Thus, growing ruzigrass also decreases P resupply from soil solid phase, resulting in P depletion in the soil solution, and a consequently lower R ratio, regardless of P rates (Table 3). At 20–40 cm, a diffusive only case was observed, which is characterized by a high initial flux into the DGT, followed by a sharp decrease of R value due to the lack of P resupply from the soil solid phase (Harper et al., 2000), but only the fallowed soil receiving 13 and 26 kg ha^−1^ of P had a constant and small supply of P in 2015 (Fig. 3). As observed by Santner et al. (2015), when P sorption sites are less occupied, as in the 20–40 cm of soil depth, the remaining sites might act as sinks, reducing the diffusion and slowing desorption.

**Table 3:** Ratio (R) of measured P_DGT_ concentration to the P_DET_, and relative resupply from solid phase (R-R_diff_) in four soil depths with ruzigrass and different P application rates, in 2014 and 2015.

Off-season	P rates (kg ha^−1^)			Off-season	P rates (kg ha^−1^)		
	0	13	26		0	13	26
2014				2015			
R (P_DGT_/P_DET_)							
0–5 cm							
Ruzigrass	0.58 ^ns^	0.48 ^ns^	0.60 ^ns^	Ruzigrass	0.35 ^ns^	0.54 ^ns^	0.37 ^ns^
Fallow	0.68 ^ns^	0.49 ^ns^	0.47 ^ns^	Fallow	0.35 ^ns^	0.36 ^ns^	0.42 ^ns^
5–10 cm							
Ruzigrass	0.07 Ab	0.04 Bb	0.22 Ba	Ruzigrass	0.07 Bb	0.11 Bb	0.25 Ba
Fallow	0.06 Ab	0.15 Ab	0.32 Aa	Fallow	0.19 Ab	0.32 Aa	0.42 Aa
10–20 cm							
Ruzigrass	0.19 Bab	0.10 Bb	0.34 Aa	Ruzigrass	0.08 Bb	0.07 Bb	0.16 Ba
Fallow	0.40 Aa	0.35 Aa	0.25 Aa	Fallow	0.16 Ab	0.23 Ab	0.33 Aa
20–40 cm							
Ruzigrass	0.04 Aa	0.03 Bb	0.05 Aa	Ruzigrass	0.07 Ba	0.06 Ba	0.09 Aa
Fallow	0.06 Ab	0.08 Aa	0.04 Ab	Fallow	0.17 Ab	0.22 Aa	0.08 Ac

R-R_diff_							
0–5 cm							
Ruzigrass	0.56 ^ns^	0.46 ^ns^	0.58 ^ns^	Ruzigrass	0.33 ^ns^	0.52 ^ns^	0.35 ^ns^
Fallow	0.66 ^ns^	0.47 ^ns^	0.45 ^ns^	Fallow	0.33 ^ns^	0.33 ^ns^	0.40 ^ns^
5–10 cm							
Ruzigrass	0.05 Ab	0.02 Bb	0.20 Ba	Ruzigrass	0.05 Bb	0.09 Bb	0.23 Ba
Fallow	0.04 Ab	0.14 Ab	0.30 Aa	Fallow	0.17 Ab	0.30 Aa	0.40 Aa
10–20 cm							
Ruzigrass	0.17 Bab	0.08 Bb	0.32 Aa	Ruzigrass	0.06 Bb	0.06 Bb	0.14 Ba
Fallow	0.38 Aa	0.33 Aa	0.24 Aa	Fallow	0.14 Ab	0.21 Ab	0.31 Aa
20–40 cm							
Ruzigrass	0.02 Aa	0.01 Bb	0.03 Aa	Ruzigrass	0.05 Ba	0.04 Ba	0.07 Aa
Fallow	0.04 Ab	0.06 Aa	0.02 Ab	Fallow	0.15 Ab	0.20 Aa	0.06 Ac

^a^ Averages followed by different lowercase letters in the line and uppercase in the column are significantly different according to *t*-test (*p* < 0.05). ^ns^ Not significant.

The relative contribution of diffusion from the soil solution (R_diff_) was very low, averaging only 0.02, and had little variation across the treatments due to the similarity of porosity and particle concentration, similar to what was observed by Mason et al. (2010). Consequently, the relative resupply from solid phase (R-R_diff_) was very similar to the total R value (Table 3). This means that the resupply from the solid phase was the main process affecting P_DGT_ concentration, which is clearly observed in Fig. 2.

The P diffusion in soils and consequently P availability is affected by soil moisture, bulk density, mineralogy, P concentration in the soil solution, and microbial activity (Barber, 1995; Hinsinger et al., 2011). The high P adsorption by Fe and Al oxides in this highly weathered soil (Almeida et al., 2018b), results in low P_DET_ and decreases diffusion flux, affecting P movement towards plant roots (Raghothama and Karthikeyan, 2005). Soil microorganisms may reduce P availability by immobilization of P in the microbial biomass (Schachtman et al., 1998). However, MBP is not related to the low P_DET_, mainly in the soil cultivated with ruzigrass (Table 4). The increase in P application rate results in an increase of ruzigrass growth and uptake of P (Almeida et al., 2018c), competing with soil microorganisms for P, indicating that P is a limiting nutrient for the microbes when ruzigrass is grown (Nziguheba et al., 1998), and not the other way around. In the fallow soil, there was probably a low competition for P between plants and microorganisms. Thus, the overall trend was opposite in ruzigrass and fallowed soils. With ruzigrass the MBP was highest at no P fertilizer application whereas to the fallowed soil the MPB increases with increasing P fertilizer application, as it would be normally expected (Table 4). It is generally assumed that rhizosphere microbes will have access to soil solution P before the plants (Menezes-Blackburn et al., 2016a). The lower MBP in soil receiving P applications and with ruzigrass would indicates that microbial proliferation is suppressed by ruzigrass. Many rhizobacteria P mobilization mechanisms have been previously described (George et al., 2017; Menezes-Blackburn et al., 2013) and the ruzigrass conditioning of the rhizosphere microbial community may have well been the indirect cause of the observed lower desorption kinetics. This hypothesis is nevertheless very speculative and needs to be further explored in future studies for the effect of ruzigrass on the abundance and activity of rhizosphere microbes, e.g. phosphobacteria.

**Table 4:** Microbial biomass phosphorus (MBP), in four soil depths with ruzigrass and different P application rates, in 2014 and 2015.

Off-season	P rates (kg ha^−1^)			Off-season	P rates (kg ha^−1^)		
	0	13	26		0	13	26
2014				2015			
MBP (mg kg^−1^)							
0–5 cm							
Ruzigrass	2.39 Aa	1.26 Ab	0.77 Ab	Ruzigrass	2.27 Aa	1.75 Ab	0.78 Ab
Fallow	1.43 Ba	1.31 Aa	1.12 Aa	Fallow	1.17 Ba	1.58 Aa	0.78 Aa
5–10 cm							
Ruzigrass	0.88 Ab	1.28 Ab	2.85 Aa	Ruzigrass	1.13 Ab	1.48 Aa	1.60 Aa
Fallow	1.08 Ab	1.78 Ab	3.72 Aa	Fallow	0.96 Ab	1.37 Aa	1.55 Aa
10–20 cm							
Ruzigrass	0.37 Ab	0.78 Aa	0.27 Bb	Ruzigrass	0.48 Aa	0.47 Aa	0.25 Bb
Fallow	0.35 Ab	0.57 Ba	0.64 Aa	Fallow	0.36 Bb	0.30 Bb	0.69 Aa
20–40 cm							
Ruzigrass	0.25 ^ns^	0.29 ^ns^	0.23 ^ns^	Ruzigrass	0.13 Bb	0.27 Aa	0.24 Aa
Fallow	0.28 ^ns^	0.23 ^ns^	0.29 ^ns^	Fallow	0.31 Aa	0.17 Bc	0.22 Ab

^a^ Averages followed by different lowercase letters in the line and uppercase in the column are significantly different according to t-test (*p* < 0.05). ^ns^ Not significant.

The kinetics of P resupply from the soil solid phase to solution can be estimated by response time, T_c_, and the rate constant, k_−1_ (Mason et al., 2010; Zhang et al., 1998). The estimated T_c_ by DIFS model can be considered as the time taken by the components of K_d_ to reach 63% of their equilibrium values after soil solution P has been depleted to zero (Honeyman and Santschi, 1988), and according to the obtained DIFS outputs, growing ruzigrass results in a longer time for the system to reach equilibrium (Table 5). According to Heidari et al. (2017), P uptake is highly correlated to soil P desorption constant. Thus, the lower k_−1_ (Table S1) and higher T_c_ (Table 5) observed after ruzigrass than fallow also results in a lower soil P availability, which is in agreement with the observed by Almeida et al. (2018b) in soil cropped to ruzigrass than fallow in the soybean off-season. The long term use of ruzigrass may have resulted in an increase of adsorption sites, due to interactions of Fe and Al oxides with the higher SOM contents (Almeida et al., 2018b), causing a higher P adsorption and a lower P resupply from solid phase. The adsorption of organic functional groups onto iron oxides can promote anion adsorption via cation bridges, and increase specific surface area by inhibiting mineral crystallization and resulting in amorphous minerals (Fink et al., 2016; Schwertmann, 1991).

Unlike the observed increase in soil P availability in other studies, using different cover crops, such as white-lupin (*Lupinus albus* L.) (Braum and Helmke, 1995) and oat (*Avena sativa* L.) (Redel et al., 2007), the use of ruzigrass as a cover crop in the off-season decreased soil P availability as shown by the lower P_DGT_ values. The use of DGT technique provided new insights into P availability in soils, by considering the P diffusion and resupply process, which have not been considered in previous studies. Inconsistencies were observed between a high P availability as determined by other soil P extraction methods, and low cash-crop yields after ruzigrass (Almeida et al., 2018c; Janegitz et al., 2016; Merlin et al., 2013). Therefore, DGT technique is a promising tool for providing a more accurate measurement of P availability to soybean plants, in soybean-ruzigrass cropping systems.

**Table 5:** System response time (T_c_) in four soil depths with ruzigrass and different P application rates, in 2014 and 2015.

Off-season	P rates (kg ha^−1^)			Off-season	P rates (kg ha^−1^)		
	0	13	26		0	13	26
2014				2015			
T_c_ (s^−1^)							
0–5 cm							
Ruzigrass	7.8E + 01 Ab^a^	1.4E + 02 Aa	5.9E + 01 Bb	Ruzigrass	3.1E + 02 Aa	7.2E + 01 Bb	2.7E + 02 Aa
Fallow	3.2E + 01 Bb	1.2E + 02 Aa	1.4E + 02 Aa	Fallow	3.0E + 02 Aa	2.9E + 02 Aab	1.7E + 02 Ab
5–10 cm							
Ruzigrass	1.1E + 04 Aab	4.2E + 04 Aa	8.8E + 02 Ab	Ruzigrass	5.4E + 03 Aa	5.3E + 03 Aa	7.5E + 02 Ab
Fallow	1.9E + 04 Aa	1.9E + 03 Bb	5.8E + 02 Ab	Fallow	1.4E + 03 Ba	3.8E + 02 Ba	1.9E + 02 Aa
10–20 cm							
Ruzigrass	1.2E + 03 Ab	4.0E + 03 Aa	2.8E + 02 Ac	Ruzigrass	7.0E + 03 Aa	8.5E + 03 Aa	1.6E + 03 Ab
Fallow	1.8E + 02 Bb	2.7E + 02 Bab	5.7E + 02 Aa	Fallow	1.7E + 03 Ba	6.8E + 02 Ba	2.8E + 02 Aa
20–40 cm							
Ruzigrass	9.0E + 04 Ab	3.7E + 05 Aa	3.0E + 04 Ac	Ruzigrass	1.8E + 04 Aa	2.1E + 04 Aa	8.2E + 03 Ab
Fallow	2.3E + 04 Bab	1.1E + 04 Bb	4.6E + 04 Aa	Fallow	2.2E + 03 Bb	1.0E + 03 Bb	1.2E + 04 Aa

a Averages followed by different lowercase letters in the line and uppercase in the column were significantly different (Tukey, *p* < 0.05).

## 4 Conclusion

The long term use of ruzigrass as cover crop in the soybean off-season decreased P mobility regardless of P rates by reducing P diffusion and resupply from the soil solid phase, in contrast to fallow, accepting the hypothesis that growing ruzigrass reduces P availability to soybean. Under ruzigrass treatment, soil solution P cannot be sustained at the same levels of fallowed soils during the continuous P depletion generated by DGTs (and roots), even at higher solid-to-solution P concentration gradients. This indicates that ruzigrass may have caused more P to be retained in the soil solid phase compared to the fallow treatment. Depletion induced P resupply from the soil solid phase to solution is the main mechanism affecting P availability in the studied soil. Therefore, long-term soybean ruzigrass crop rotation resulted in a lower soil P availability, which may have a significant impact on crop production.

The following is the supplementary data related to this article.

Table S1 Phosphorus concentration in soil solution phase (P_DET_), effective soil phosphorus concentration (P_E_), and desorption rate constant (k_−1_) in four soil depths with ruzigrass and different P application rates, in 2014 and 2015. Table S1
