# Supplementary material for: Phosphorus availability and dynamics in soil affected by long-term ruzigrass cover crop
Source: Geoderma. 2019 Mar 1;337:434–43. doi: 10.1016/j.geoderma.2018.09.056 (PMC6358123; doi:10.1016/j.geoderma.2018.09.056)
Supplement: Table S1 — Phosphorus concentration in soil solution phase (PDET), effective soil phosphorus concentration (PE), and desorption rate constant (k−1) in four soil depths with ruzigrass and different P application rates, in 2014 and 2015. [file mmc1.docx]

# Table S1. Phosphorus concentration in soil solution phase (P_DET_), effective soil phosphorus concentration (P_E_), and desorption rate constant (k_-1_) in four soil depths with ruzigrass and different P application rates, in 2014 and 2015.

| Off-season | | P rates (kg ha^-1^) | | | | | | Off-season | | P rates (kg ha^-1^) | | | | |  |
| --- | --- | --- | --- | --- | --- | --- | --- | --- | --- | --- | --- | --- | --- | --- | --- |
|  |  | 0 | | 13 | | 26 | |  |  | 0 | | 13 | 26 | |  |
| 2014 | | | | | | | | 2015 | | | | | | |  |
| ----------------------------------------------------------- P_DET_ (µg L^-1^) ----------------------------------------------------------- | | | | | | | | | | | | | | |  |
| 0–5 cm | | | | | | | | | | | | | | |  |
| Ruzigrass | | 7.4 Ab^a^ | | 29.1 Aa | | 28.6 Aa | | Ruzigrass | | 26.3 Ab | | 40.7 Aab | 56.6 Aa | |  |
| Fallow | | 13.4 Ab | | 28.0 Aa | | 36.5 Aa | | Fallow | | 36.2 Ab | | 50.4 Aab | 55.9 Aa | |  |
| 5–10 cm | | | | | | | | | | | | | | |  |
| Ruzigrass | | 11.7 Bb | | 19.4 Aab | | 25.4 Ba | | Ruzigrass | | 12.3 Ab | | 13.1 Bb | 26.2 Ba | |  |
| Fallow | | 21.1 Ab | | 17.0 Ab | | 40.1 Aa | | Fallow | | 12.1 Ab | | 19.5 Ab | 31.7 Aa | |  |
| 10–20 cm | | | | | | | | | | | | | | |  |
| Ruzigrass | | 6.0 Ab | | 11.1 Aab | | 16.5 Aa | | Ruzigrass | | 8.7 Aa | | 9.6 Aa | 11.0 Ba | |  |
| Fallow | | 8.3 Ab | | 8.7 Ab | | 19.5 Aa | | Fallow | | 5.9 Bb | | 8.2 Ab | 24.0 Aa | |  |
| 20–40 cm | | | | | | | | | | | | | | |  |
| Ruzigrass | | 18.2 Aab | | 19.2 Aa | | 14.6 Ab | | Ruzigrass | | 9.9 Ab | | 14.6 Aa | 11.1 Bb | |  |
| Fallow | | 17.2 Aa | | 13.1 Ba | | 16.7 Aa | | Fallow | | 8.8 Ab | | 7.4 Bb | 19.5 Aa | |  |
| ----------------------------------------------------------- P_E_ (mg L^-1^) ----------------------------------------------------------- | | | | | | | | | | | | | | |  |
| 0–5 cm | | | | | | | | | | | | | | |  |
| Ruzigrass | | 0.25 Ac | | 0.58 Ab | | 0.77 Aa | | Ruzigrass | | 0.43 Ab | | 0.96 Aa | 1.10 Aa | |  |
| Fallow | | 0.41 Ac | | 0.60 Ab | | 0.92 Aa | | Fallow | | 0.52 Ac | | 0.83 Ab | 1.08 Aa | |  |
| 5–10 cm | | | | | | | | | | | | | | |  |
| Ruzigrass | | 0.06 Ab | | 0.04 Ab | | 0.31 Ba | | Ruzigrass | | 0.04 Bb | | 0.06 Bb | 0.60 Ba | |  |
| Fallow | | 0.18 Ab | | 0.16 Ab | | 0.78 Aa | | Fallow | | 0.11 Ab | | 0.30 Ab | 1.02 Aa | |  |
| 10–20 cm | | | | | | | | | | | | | | |  |
| Ruzigrass | | 0.04 Ab | | 0.06 Bb | | 0.15 Ba | | Ruzigrass | | 0.03 Ab | | 0.04 Bb | 0.10 Ba | |  |
| Fallow | | 0.06 Ab | | 0.15 Ab | | 0.35 Aa | | Fallow | | 0.05 Ac | | 0.11 Ab | 0.44 Aa | |  |
| 20–40 cm | | | | | | | | | | | | | | |  |
| Ruzigrass | | 0.03 Ba | | 0.02 Bb | | 0.04 Aa | | Ruzigrass | | 0.03 Bb | | 0.04 Ba | 0.05 Ba | |  |
| Fallow | | 0.04 Aab | | 0.05 Aa | | 0.03 Ab | | Fallow | | 0.06 Ab | | 0.08 Aa | 0.07 Aa | |  |
| ---------------------------------------------------------------- k_–1_ (s^-1^) ---------------------------------------------------------------- | | | | | | | | | | | | | | | |
| 0–5 cm | | | | | | | | | | | | | | | |
| Ruzigrass | 1.7E-06 Bb | | 2.5E-06 Aab | | 4.3E-06 Aa | | Ruzigrass | | 1.6E-06 Ab | | 6.0E-06 Aa | | | 1.8E-06 Ab | |
| Fallow | 6.4E-06 Aa | | 2.7E-06 Ab | | 2.5E-06 Ab | | Fallow | | 1.6E-06 Aa | | 2.0E-06 Bb | | | 3.2E-06 Aa | |
| 5–10 cm | | | | | | | | | | | | | | | |
| Ruzigrass | 4.0E-08 Ab | | 1.3E-08 Bb | | 3.4E-07 Ba | | Ruzigrass | | 7.2E-08 Aa | | 6.7E-08 Ba | | | 3.0E-07 Ba | |
| Fallow | 3.9E-08 Ab | | 2.1E-07 Ab | | 1.1E-06 Aa | | Fallow | | 3.0E-07 Ac | | 1.2E-06 Ab | | | 1.9E-06 Aa | |
| 10–20 cm | | | | | | | | | | | | | | | |
| Ruzigrass | 1.8E-07 Bb | | 1.2E-07 Bb | | 1.6E-06 Aa | | Ruzigrass | | 2.8E-08 Aa | | 3.1E-08 Ba | | | 1.0E-07 Ba | |
| Fallow | 2.0E-06 Aa | | 1.2E-06 Ab | | 9.3E-07 Ab | | Fallow | | 9.3E-08 Ac | | 5.7E-07 Ab | | | 2.1E-06 Aa | |
| 20–40 cm | | | | | | | | | | | | | | | |
| Ruzigrass | 2.1E-08 Ba | | 5.0E-09 Bb | | 3.4E-08 Aa | | Ruzigrass | | 3.7E-08 Bb | | 5.9E-08 Bab | | | 1.3E-07 Aa | |
| Fallow | 5.5E-08 Ab | | 1.0E-07 Aa | | 3.0E-08 Ab | | Fallow | | 3.1E-07 Ab | | 6.4E-07 Aa | | | 1.4E-07 Ac | |

^a^ Averages followed by different lowercase letters in the line and uppercase in the column are significantly different according to t-test (*p* < 0.05).
